# Association between Single Nucleotide Polymorphisms Related to Vitamin D Metabolism and the Risk of Developing Asthma

**DOI:** 10.3390/nu15040823

**Published:** 2023-02-05

**Authors:** Susana Rojo-Tolosa, Laura Elena Pineda-Lancheros, José María Gálvez-Navas, José Antonio Sánchez-Martínez, María Victoria González-Gutiérrez, Andrea Fernández-Alonso, Concepción Morales-García, Alberto Jiménez-Morales, Cristina Pérez-Ramírez

**Affiliations:** 1 Respiratory Medicine Department, University Hospital Virgen de las Nieves, 18014 Granada, Spain; 2 Pharmacogenetics Unit, Pharmacy Service, University Hospital Virgen de las Nieves, 18014 Granada, Spain; 3 Biomedical Research Center, Department of Biochemistry and Molecular Biology II, Institute of Nutrition and Food Technology “José Mataix”, University of Granada, Avda. del Conocimiento s/n., 18016 Granada, Spain; 4 Cancer Registry of Granada, Andalusian School of Public Health, Carretera del Observatorio 4, 18011 Granada, Spain

**Keywords:** asthma, risk, vitamin D metabolic pathway, *CYP27B1*, *CYP2R1*, GC, *CYP24A1*, *VDR*, single nucleotide polymorphisms, biomarker

## Abstract

Asthma is a chronic non-communicable disease that affects all age groups. The main challenge this condition poses is its heterogeneity. The role of vitamin D in asthma has aroused great interest, correlating low vitamin D levels and polymorphisms in the genes involved in its metabolic pathway with the risk of asthma. The aim of this study was to evaluate the influence of 13 single nucleotide polymorphisms (SNPs) related to the vitamin D metabolism on the susceptibility to asthma. An observational case-control study was performed, including 221 patients with asthma and 442 controls of Caucasian origin from southern Spain. The SNPs *CYP24A1* (rs6068816, rs4809957), *CYP27B1* (rs10877012, rs4646536, rs703842, rs3782130), *GC* (rs7041), *CYP2R1* (rs10741657) and *VDR* (ApaI, BsmI, FokI, Cdx2, TaqI) were analyzed by real-time PCR, using TaqMan probes. The logistic regression model adjusted for body mass index revealed that in the genotype model, carriers of the Cdx2 rs11568820-AA genotype were associated with a higher risk of developing asthma (*p* = 0.005; OR = 2.73; 95% CI = 1.36–5.67; AA vs. GG). This association was maintained in the recessive model (*p* = 0.004). The haplotype analysis revealed an association between the ACTATGG haplotype and higher risk of asthma for the rs1544410, rs7975232, rs731236, rs4646536, rs703842, rs3782130 and rs10877012 genetic polymorphisms (*p* = 0.039). The other SNPs showed no effect on risk of developing asthma. The Cdx2 polymorphism was significantly associated with the susceptibility of asthma and could substantially act as a predictive biomarker of the disease.

## 1. Introduction

Asthma is a chronic inflammatory disease of the airways whose physiological response is bronchial hyperreactivity and that clinically takes the form of repeated episodes of dyspnea, wheezing and flare-ups. These episodes are usually associated with airflow obstruction, partially or totally reversible spontaneously or with specific treatment [1].

Asthma is a chronic non-communicable disease that affects all age groups. According to the Global Burden of Disease study 2015, the global prevalence of asthma increased by 12.6% from 1990 to 2015 [2]. It is estimated that 300 million people worldwide suffer from asthma, and it is the most common chronic disease, responsible for significant morbidity and mortality [3]. The exponential growth in asthma diagnosis in recent decades, followed by the World Health Organization’s estimate of 100 million additional asthmatics by 2025, make it a worldwide public health problem [4].

The main challenge this condition poses is its heterogeneity. Numerous types of risk factors positively associated with the presence of the asthmatic syndrome can be distinguished: host factors (rhinitis, bronchial hyperresponsiveness, obesity, atopy), perinatal factors (preeclampsia, prematurity, smoking during pregnancy, caesarean section, mother’s diet), environmental factors (allergens, tobacco, pollution), medications and genetic factors [5].

Vitamin D function in asthma has aroused great interest, correlating low vitamin D levels and polymorphisms in the genes involved in its metabolic pathway with the risk of asthma [6,7]. Vitamin D and its receptor (VDR) have immunomodulatory and anti-inflammatory properties, dampening the effects of the immune response, both innate and adaptive, due to their effect on dendritic cells, macrophages and T lymphocytes (CD4 + CD8) [8]. Vitamin D also acquires an important role in airway remodeling through changes in epithelial cells and alveolar macrophages and transcription of pro-inflammatory cytokines [3,8]. The active isoform of vitamin D, 1α,25-Dihydroxyvitamin D3 (1,25(OH)2D3), has a direct effect on the development of helper (Th) cells, inhibiting Th1 and augmenting Th2 cell development, which entails an increase in IL-4, IL-5 and IL-10 production [9]. Moreover, the gene that codes for the vitamin D binding protein (*GC*) has also been described as a factor in susceptibility to developing asthma [7].

Vitamin D has a complex metabolism with enzymes that regulate its active (1,25(OH)2D) and inactive (25(OH)D) forms and its catabolism. Various cytochrome P450 (CYP) enzymes are responsible for the balance between the inactive and active forms of vitamin D: CYP2R1 (25-hydroxylase), responsible for the first hydroxylation that leads to the inactive form 25(OH)D, CYP27B1 (1-α-hydroxylase), which generates 1,25(OH)2D through its 1-hydroxylase activity, and CYP24A1 (24-hydroxylase), which catalyzes both 25(OH)D and 1,25(OH)2D. The 1,25(OH)2D, is a secosteroid hormone which acts as a ligand by selectively binding to the VDR receptor, a transcription factor which binds to DNA sites called vitamin D response elements (VDREs). There are thousands of VDREs regulating hundreds of genes specifically for each cell [10,11,12,13,14]. In this conceptual framework, vitamin D plays an important role in the inflammatory response and the development of the Th2 phenotype, typical of asthmatic disease.

The *VDR* gene is highly polymorphic. It is located at chromosome 12q13.11 and numerous extensively studied single-nucleotide polymorphisms (SNPs) have been described for it, including ApaI (rs7975232, intron 8, 64978 C > A), BsmI (rs1544410, intron 8, 63980 G > A), TaqI (rs731236, exon 9, 65058 T > C) FokI (rs2228570, exon 2, 30920 C > T) and Cdx2 (rs11568820, intron 1, +1270 G > A) [15,16,17]. BsmI, ApaI and TaqI can modify the expression of the *VDR* gene without producing a structural change in the protein, due to their position in the three prime untranslated region (3′ UTR), which is related to the stability of the mRNA of *VDR* [10,18,19]. FokI is located in the start codon, producing a change from thymine to cytosine, and this results in two different translation initiation sites, generating two variants of the VDR protein, one long and the other short [20]. The FokI SNP is associated with variations in vitamin D levels, indicating important consequences in its metabolism [18,21]. The *GC* gene, which shows a high degree of polymorphism, is at chromosome 4q13, with an SNP characteristically associated with asthma (rs7041) in codon 420 [7].

Vitamin D precursors need to be hydroxylated in order to be activated. Firstly, a hydroxylation takes place at position 25 in the liver, performed by the CYP2R1 or 25-hydroxylase enzyme (encoded by the gene CYP2R1). As a result of this reaction, calcidiol is obtained [22,23]. The second hydroxylation needed to activate vitamin D takes place in the kidney, by means of the CYP27B1 or 1-α-hydroxylase enzyme [24,25]. Finally, the vitamin D molecule is inactivated by the CYP24A1 or 24-hydroxylase enzyme [26,27].

Previous studies have evaluated the association between these variants of the *VDR* gene and the risk of suffering from asthma. However, the results are contradictory [7,28,29,30,31,32,33,34,35,36,37].

Taking the background presented into consideration, we conducted this study with the aim of evaluating the effects of SNPs in the genes involved in the vitamin D pathway (*CYP27B1*, *CYP24A1*, *GC* and *CYP2R1*) and in the gene encoding its receptor, *VDR* (ApaI, BsmI, FokI, TaqI and Cdx2), on the risk of developing asthma.

## 2. Materials and Methods

We conducted an observational case-control study.

### 2.1. Study Subjects

This study, with a case/control ratio 1:2, included 221 patients with asthma and 442 controls of Caucasian origin from southern Spain. The cases were recruited at the Hospital Universitario Virgen de las Nieves (Granada, Spain) from March 2013 to April 2022. The controls were individuals over the age of 18 who were recruited at the same hospital and had been living in the same geographical area, with no personal history of asthma.

This case-control study was approved by the Ethics Committee of the Sistema Andaluz de Salud (Andalusian Health Service) and conducted in accordance with the Declaration of Helsinki (code: 2112-N-22). An informed consent form for the donation of blood and saliva samples to the biobank was signed by all the subjects participating in the study. The samples received a confidential codification and treatment.

### 2.2. Socio-Demographic and Clinical Variables

The socio-demographic and clinical data included were sex, age, smoking status, previous COVID-19 infection, body mass index (BMI), allergies, daily dose of inhaled corticosteroids (ICS), need for oral corticosteroids (OCS), lung function, presence of exacerbations and blood eosinophil count. Each individual’s smoking status was defined as follows: active smoker (consumption of 100 cigarettes or more in life and currently smoke), former smoker (consumption of 100 cigarettes or more in life but currently do not smoke) and non-smoker (no cigarette consumption in life or consumption of fewer than 100 cigarettes in life). Individuals were classified by BMI range following the criteria of the Sociedad Española para el Estudio de la Obesidad (Spanish Society for the Study of Obesity): underweight (BMI ≤ 18.5), normal weight (18.5 < BMI < 24.9), overweight (25 < BMI < 29.9), obese (BMI ≥ 30) [38]. The criteria for assessing asthma were based on the Guía Española para el Manejo del Asma (Spanish Asthma Management Guidelines) (GEMA 5.2) and the Global Initiative for Asthma (GINA) [5,39]. Allergies, OCS and exacerbations were evaluated as present or absent (Yes/No) and ICS as milligrams per day. To evaluate lung function the maximum percentage expiratory volume in the first second of forced exhalation (%FEV1) was used, and blood eosinophil count was analyzed as cells per microliter. All sociodemographic and clinical variables mentioned in the cases were collected at the time of diagnosis.

### 2.3. Genetic Variables

#### 2.3.1. DNA Isolation

The Biobank of the University Hospital Virgen de las Nieves, which is part of the Andalusian Health Service, granted the DNA samples, isolated form saliva or blood. The saliva samples were collected in BD Falcon 50 mL conical tubes (BD, Plymouth, United Kingdom). The blood samples were collected in BD Vacutainer tubes with EDTA K3 as anticoagulant (3 mL). DNA extraction was performed using the QIAamp DNA Mini extraction kit (Qiagen GmbH, Hilden, Germany), following the specifications provided by the manufacturer for purification of DNA from saliva or blood, and stored at −40 °C. NanoDrop 2000 UV spectrophotometer with 280/260 and 280/230 absorbance ratios were used to measure DNA concentration and purity.

#### 2.3.2. Genotyping and Quality Control

The SNPs were determined by real-time polymerase chain reaction (PCR) allelic discrimination assay using TaqMan probes (ABI Applied Biosystems, 7300 Real-Time PCR System, 96 wells), following the manufacturer’s instructions (Table 1). The *VDR* BsmI (rs1544410), *CYP27B1* rs703842 and *CYP27B1* rs3782130 polymorphisms were analyzed using a custom assay by ThermoFisher Scientific (Waltham, MA, United States), coded as AN324M4, ANH6J3F and ANGZRHH, respectively. Sanger sequencing was used in ten percent of the samples to confirm the results obtained. Sanger sequencing and Real-time PCR were performed in the Pharmacogenetics Unit of the Hospital Universitario Virgen de las Nieves. The criteria for SNP quality control were: (1) missing genotype rate per SNP < 0.05, (2) minor allele frequency > 0.01, (3) *p* value > 0.05 in Hardy-Weinberg equilibrium test and (4) missing genotype rate between cases and controls < 0.05.

#### 2.3.3. Statistical Analysis

The cases and controls were paired based on sex and age using the propensity score matching method (1:2) [40]. The quantitative variables were expressed as the mean (±standard deviation) for those that complied with normality and as the median and percentiles (25th and 75th) for the variables that did not follow a normal distribution. Normality was confirmed with the Kolmogorov–Smirnov test.

We determined the Hardy–Weinberg equilibrium, the haplotype frequencies and the linkage disequilibrium (LD) through the D’ and r2 coefficients. The bivariate analysis of association between the risk of asthma and the polymorphisms was performed with multiple models (genotypic, recessive, dominant, allelic and additive) using the Pearson χ^2^ test and the Fisher exact test to calculate the odds ratio (OR) and the 95% confidence interval (CI). The models were defined by Purcell et al. in the PLINK tool set [41]. The Bonferroni correction was used for multiple comparisons. We considered unconditional multiple logistic regression models (genotypic, recessive and dominant) to determine the influence of possible confounding variables on the risk of suffering from asthma. The multivariate logistic regression model was performed including all the variables that were significant in the bivariate logistic regression model. The final model was adjusted only with the variables that maintained statistical significance. All the tests were 2-sided, with a significance level of *p* < 0.05, and were carried out using PLINK and the R 4.2.0 statistical program [41,42]. Linkage disequilibrium was performed with Haploview 4.2 software and the haplotype analysis with SNPStats [43,44].

## 3. Results

### 3.1. Patient Characteristics

A total of 663 individuals of Caucasian origin were included in the study: 221 asthma cases and 442 controls. Their socio-demographic, clinical and pathological characteristics are described in Table 2.

In the group of cases, the median age was 56 (46,66) years and 66.5% (147/221) were women. With regard to smoking status, 71.5% (158/221) were non-smokers, 22.6% (50/221) former smokers and 5.9% (13/221) smokers. Most of the patients were obese or overweight, 41.1% (86/209) and 37.8% (79/209), respectively. The control group had a median age of 60 (51,67) years and 62.2% (275/442) were women. A total of 51.4% (225/442) had never smoked, 21.7% (95/442) were former smokers and 29.9% (118/442) were current smokers. As for BMI, a high percentage were overweight, 36.2% (113/313), and 30.8% (96/313) of patients were obese. A total of 8.3% (34/412) had had COVID before the study.

There were statistically significant differences between the cases and the controls with regard to smoking status (*p* < 0.001; OR = 0.16; 95% CI = 0.08–0.28; Current smoker vs. non-smoker and *p* < 0.001; OR = 0.75; 95% CI = 0.50–1.11; Former smoker vs non-smoker), BMI (*p* = 0.006; OR = 2.1; 95% CI = 1.33–3.33; obese vs normal weight and *p* = 0.006; OR = 1.64; 95% CI = 1.04–2.59; overweight vs normal weight), No statistically significant differences were observed in sex (*p* = 0.278), age (*p* = 0.091) and COVID-19 (*p* = 0.963).

### 3.2. Genotype Distribution

**Figure 1:**
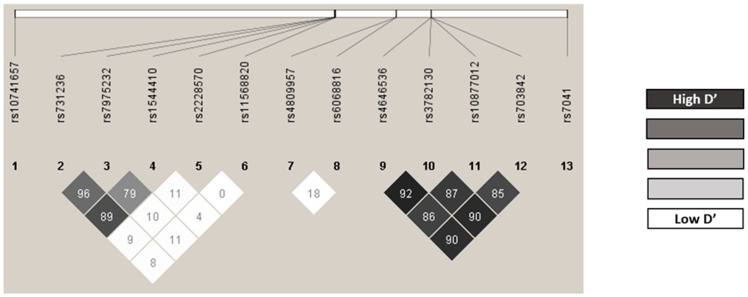
Linkage disequilibrium (LD). Polymorphisms with strong linkage disequilibrium are shown in darker shades and those with less linkage disequilibrium are shown in lighter shades.

The observed genotype frequencies were in line with the expected values according to the Hardy-Weinberg equilibrium, except *CYP24A1* rs4809957 for the control group (*p* = 0.034, Table S1). No statistical differences were found from those described in the Iberian population for this variant (*CYP24A1* rs4809957 allele G: 0.224 vs. 0.215; *p* = 0.987) [45]. The D’ and r2 LD values are shown in Table S2 and Figure 1 shows the LD graph. The following pairs of polymorphisms showed strong linkage disequilibrium: *CYP27B1* rs4646536/rs3782130 (r2 = 0.80826; D’ = 0.92497), *CYP27B1* rs4646536/rs10877012 (r2 = 0.67365; D’ = 0.86887), *CYP27B1* rs4646536/rs703842 (r2 = 0.75451; D’ = 0.87413), *CYP27B1* rs3782130/rs10877012 (r2 = 0.70678; D’ = 0.87413) and *CYP27B1* rs3782130/rs703842 (r2 = 0.79847; D’ = 0.90819). All the polymorphisms showed a minor allele frequency greater than 1%, and therefore none of them was excluded for the analysis (Table S3). The estimated haplotype frequencies are presented in Table S4.

### 3.3. Influence of Gene Polymorphisms on the Risk of Asthma

The bivariate analysis was performed taking account of the genotypic, dominant, recessive, additive and allelic, models for all the polymorphisms and the risk of developing asthma (Table S5). The *VDR* Cdx2 (rs11568820) gene polymorphism was the only one that showed a significant association for the genotypic (*p* = 0.004), recessive (*p* < 0.001), allelic (*p* = 0.045) and additive (*p* = 0.044) models (Table S5). After correction by the Bonferroni method, the genotypic (*p* = 0.039) and recessive (*p* = 0.011) models maintained a significant association with the risk of developing asthma (Table S5). For the genotypic model, patients carrying the *VDR* Cdx2 (rs11568820) AG genotype showed a higher risk of developing asthma (p_Bonferroni_ = 0.039; OR = 2.76; 95% CI = 1.46–5.29, AG vs. GG, Table 3). Similarly, carriers of the *VDR* Cdx2 (rs11568820) AA genotype also showed a higher risk of developing asthma (p_Bonferroni_ = 0.039; OR = 2.67; 95% CI = 1.43–5.04, AA vs. GG, Table 3). Likewise, for the recessive model, carriers of the *VDR* Cdx2 (rs11568820) AA genotype showed a higher risk of suffering from asthma (p_Bonferroni_ = 0.011; OR = 2.71; 95% CI = 1.48–5.02, AA vs. G, Table 3). The logistic regression model adjusted for BMI maintained the association obtained for the genotypic model (*p* = 0.005; OR = 2.73; 95% CI = 1.36–5.67; AA vs. GG, Table 4) and the recessive model (*p* = 0.004; OR = 2.73; 95% CI = 1.39–5.57; AA vs. G, Table 4). In the other polymorphisms studied, no statistically significant associations were found between any of the models analyzed and the risk of asthma (Table S5). A haplotype analysis was performed for the polymorphisms found to be in strong linkage disequilibrium (Table 5), revealing an association between the ACTATGG haplotype and increased risk of asthma for the rs1544410, rs7975232, rs731236, rs4646536, rs703842, rs3782130 and rs10877012 polymorphisms (*p* = 0.039; OR = 2.84; 95% CI = 1.06–7.64, Table 5).

## 4. Discussion

Asthma is a complex disease of multifactorial pathogenesis with marked inflammatory activity, involving Th2 cells, as well as type 2 innate lymphoid cells (ILC2) which release proinflammatory cytokines [5,46]. Vitamin D has been related to several cell processes, such as cell proliferation and differentiation, inflammation, apoptosis, etc., and especially immune system regulatory function, due to the presence of the enzymes needed for its synthesis and that of its receptor in macrophages, dendritic cells, monocytes, T and B cells and structural epithelial cells. These elements are essential parts of the immune system in its protective mission at pulmonary level [23,24,47,48]. Previous studies have discovered a genetic correlation between polymorphisms in the vitamin D metabolic pathway and asthma, due to the start of inflammatory processes and deregulation of the immune system, mainly in a pediatric population [6,7,49,50,51]. Our study was carried out in order to ascertain the influence of eight genetic polymorphisms located in the main genes that regulate vitamin D metabolism (*CYP24A1, GC, CYP27B1* and *CYP2R1*) and five SNPs in the gene that encodes its receptor (*VDR*) on the susceptibility to asthma, in a Caucasian population in southern Spain.

One of the most studied genes because of its immunomodulatory and anti-inflammatory properties is the vitamin D receptor gene [8,52]. Various studies have found a relationship between the activity of VDR and the presence, differentiation and functionality of T cells [53]. The presence of single nucleotide polymorphisms in the vitamin D receptor gene (*VDR*) may modify its expression, and, consequently, the biological function of vitamin D, influencing the emergence of asthmatic disease [7,9]. Gene polymorphisms in *VDR* have been extensively studied, the most clinically important being BsmI (rs1544410), Cdx2 (rs11568820), FokI (rs2228570) and TaqI (rs731236). The Cdx2 (rs11568820) polymorphism is located in the promoter region of the 5′ end of the *VDR* gene and may influence the correct binding of the primer and, consequently, alter the transcription [15,19]. The results of our study show a statistically significant association between the risk of asthma and the presence of the A allele in the *VDR* Cdx2 (rs11568820) polymorphism (Table 2). There are not yet any studies that positively correlate the Cdx2 polymorphism and asthma in an adult population, but it has been described in a pediatric population and in other respiratory conditions. In line with our results is a study in a mixed pediatric population (n = 60 cases/17 controls, Brazil), which shows a significant association between the presence of the AA genotype in the *VDR* Cdx2 SNP and developing asthma (*p* = 0.003; AA vs. G) [54]. A recent cohort study conducted in a mixed population (725 subjects) showed a significant association between the Cdx2 (rs11568820-AA) genotype and the susceptibility to upper respiratory tract infection (*p* = 0.001; OR = 1.31; 95% CI = 1.12–1.53; AA vs. GG) [55].

The FokI (rs2228570) polymorphism gives rise to a transcription start codon, and the FokI G variant produces a shorter form of protein, which could influence its functionality [17,18,21]. In our study, no association was found between FokI (rs2228570) and developing asthma. This finding is in line with a meta-analysis from 2014 in a mixed population (n = 1997 cases/1868 controls; China, Canada, Iran, Tunisia, Egypt and the United States) and a study in a Caucasian population (n = 110 cases/110 controls) which report no association between the risk of asthma and this polymorphism [56]. However, another recent meta-analysis from 2022, conducted in a mixed pediatric population (n = 1039 cases/894 controls; Egypt, Turkey, Chile, China, Ireland, Greece, Tunisia, Cyprus and the United States), associates the dominant and additive models of the *VDR* FokI polymorphism with a lower risk of developing asthma (*p* = 0.004; OR = 0.67; 95% CI = 0.51–0.88; I2 = 11.6%; p(I2) = 0.34; TT + CT vs CC and *p* = 0.015; OR = 0.63; 95% CI = 0.43–0.92; I2 = 62.7%; p(I2) = 0.03; T vs. C, respectively) [57].

The BsmI (rs1544410) polymorphism may affect the function of VDR in regulating the stability of mRNA and the efficiency of translation of the protein [34,37]. In this study, we found no association between the BsmI polymorphism and the risk of suffering from asthma. In line with our results is the meta-analysis by Zhou et al. (n = 947 cases/1552 controls) and a study in a Caucasian population (n = 110 cases/110 controls), where no association was found between the SNP and asthmatic disease [37,57]. In contrast, the meta-analysis by Tizaoui et al. (n = 823 cases/954 controls) associated the AA genotype with an increased risk of developing asthma (*p* = 0.017; OR = 2.017; 95% CI = 1.236–3.851; I2 = 67.5%; p(I2) = 0.05; AA vs. GG) [56].

The ApaI (rs7975232) SNP has the potential to alter the regulation of splicing [58]. In agreement with our study are the two meta-analyses, one conducted in a mixed population (n = 1478 cases/1441 controls) and the other in a mixed pediatric population (n = 1217 cases/1753 controls), which did not observe any association between the ApaI SNP and developing asthma [56,57].

The TaqI (rs731236) is a synonymous polymorphism that does not affect the sequence of amino acids, but, being located near the 3′ UTR of mRNA, it may alter the functionality of the protein [58]. In this study, we did not find a statistically significant association between this SNP and the risk of suffering from asthma. Previous studies show contradictory results. The meta-analysis by Tizaoui et al., conducted in a mixed population (n = 1478 cases/1441 controls) showed a significant association between the TaqI C allele and the risk of suffering from asthma (*p* = 0.04; OR = 1.488; 95% CI = 1.019–2.174; I2 = 30.21%; p(I2) = 0.46; TC vs. CC) [56]. However, the 2022 meta-analysis in a mixed pediatric population (n = 898 cases/1428 controls) associated the presence of the *VDR* TaqI C allele as a protective factor against the presence of the condition in the additive model (*p* = 0.022; OR = 0.45; 95% CI = 0.23–0.89; I2 = 55.4%; p(I2) = 0.106; C vs. T) [57].

The *GC* gene synthesizes the vitamin D binding protein (VDBP). The rs7041 producing a change from thymine to guanine [58,59]. Our study has not demonstrated the existence of a significant association between the *GC* rs7041 polymorphism and the susceptibility to asthma. However, two studies conducted in Caucasian populations, adult (n = 110 cases/110 controls) and pediatric (n = 96 cases/96 controls), showed that individuals carrying the GG genotype had a higher risk of suffering from asthma (*p* = 0.014; OR = 2.85; 95% CI = 1.21–5.5; GG vs. TT + TG and *p* < 0.0001; OR = 10.7; 95% CI = 4.26–26.9; GG vs. TT + GT, respectively) [37,60].

In our study, the presence of *CYP2R1* rs10741657 was not related to a higher risk of suffering from asthma. Similarly, previous studies conducted in a Caucasian population (n = 154 cases/154 controls) and a Caucasian pediatric population (n = 1386 cases/1305 controls) did not observe any association between this SNP and the susceptibility to asthma [49,61].

Our study included the rs3782130, rs4646536, rs703842 and rs10877012 polymorphisms in the *CYP27B1* gene, which were not associated with the susceptibility to asthma. In line with our findings, a study in a Caucasian population (n = 154 cases/154 controls), mentioned above, found no statistically significant association between *CYP27B1* rs108777012 and the risk of asthma [61]. Moreover, a systematic review showed that the rs4646536 and rs703842 polymorphisms are not related to the risk of developing asthma [49]. To date, there are no studies determining that the *CYP27B1* rs3782130 polymorphism influences the development of asthma.

The *CYP24A1* rs6068816 and rs4809957 polymorphisms have not been related in our study to the risk of developing asthma. There are currently no studies in the literature that evaluate the association of these polymorphisms with the susceptibility to asthma.

The haplotype analysis revealed that the ACTATGG haplotype (*p* = 0.032; OR = 2.84; 95% CI: 1.06–7.64) for BsmI, ApaI, TaqI, *CYP27B1* (rs4646536), *CYP27B1* (rs703842), *CYP27B1* (rs3782130), *CYP27B1* (rs10877012) was associated with a higher risk of asthma. The presence of the TaqI and ApaI polymorphisms in specific haplotypes affects the stability of the mRNA of *VDR* and the transcription rate and may alter protein expression. In line with this, a cohort study in an Asian pediatric population (n = 143) revealed that the CA haplotype of *VDR* rs7975232/rs1544410 was associated with greater susceptibility to bronchial asthma [16].

The main limitation of this study is the small sample size compared to other available studies. This may have prevented us from detecting certain associations. However, the logistic regression analysis, where the effect described for the *VDR* Cdx2 (rs11568820) polymorphism on the risk of developing asthma was maintained, made it possible to avoid false positive associations. Another limitation of the study is that there is little information to be found in Caucasian and adult populations; most of the studies in which the presence of the polymorphisms studied is related to the risk of asthma are in Asian and paediatric populations. On the other hand, the strengths of our study lie in the homogeneity of the group of cases, composed of patients with asthma recruited in the same geographical area, as well as the homogeneity between cases and controls, which facilitates comparison between the groups.

Further studies that include a wider range of polymorphisms are needed and samples with a larger number of participants of Caucasian origin will be required to confirm or rule out the influence of these genes on the risk of developing asthma.

## 5. Conclusions

The Cdx2 (rs11568820) polymorphism was significantly associated with the susceptibility to asthma. No association was found with the other polymorphisms of the *VDR* gene, ApaI (rs7975232), BsmI (rs1544410), FokI (rs2228570) and TaqI (rs731236), nor with the polymorphisms studied in genes involved in vitamin D pathway: *CYP24A1* (rs4809957, rs6068816), *CYP2R1* (rs10741657), *GC* (rs7041) and *CYP27B1* (rs703842, rs4646536, rs10877012, rs3782130).

## Figures and Tables

**Table 1:** Gene polymorphisms and TaqMan ID.

Gene	Location, SNP	dbSNP ID	Assay ID
*VDR* (12q13.11)	Intron 8, C > A	rs7975232 (ApaI)	C__28977635_10
	Intron 8, G > A	rs1544410 (BsmI)	AN324M4
	Exon 2, C > T	rs2228570 (FokI)	C__12060045_20
	Intron 1, G > A	rs11568820 (Cdx2)	C___2880808_10
	Exon 9, T > C	rs731236 (TaqI)	C___2404008_10
*CYP27B1* (12q14.1)	3′ UTR, A > G	rs703842	ANH6J3F
	Promotor 5′, G > C	rs3782130	ANGZRHH
	5′ UTR, A > G Intron 6, T > C	rs10877012 rs4646536	C__26237740_10 C__25623453_10
*CYP24A1* (20q13.2)	Exon 6, G > A	rs6068816	C__25620091_20
	3′ UTR, G > C	rs4809957	C___3120981_20
*CYP2R1* (11p15.2)	5′ UTR, A > G	rs10741657	C___2958430_10
*GC* (4q13.3)	Exon 11, T > G	rs7041	C___3133594_30

**Table 2:** Clinical-pathological characteristics of asthma cases and controls.

	Cases		Controls		χ²	*p* Value	Reference	OR	95% CI
	N	n (%)	N	n (%)					
Gender	221		442						
Female		147 (66.5)		275 (62.2)	1.177	0.278			
Male		74 (33.5)		167 (37.8)					
Age	221	56 (46, 66)	442	60 (51, 67)		0.091 *			
Smoking status	221		442						
Current smokers		13 (5.9)		118 (29.9)	43.06	<0.001	Non-smokers	0.16	0.08–0.28
Former smokers		50 (22.6)		95 (21.7)				0.75	0.50–1.11
Non-smokers		158 (71.5)		225 (51.4)				1	
COVID-19	221		412						
Yes		18 (8.1)		34 (8.3)	0.002	0.963			
No		203 (91.9)		378 (91.7)					
BMI	209		313						
Normal weight		44 (21.1)		103 (33.0)	10.29	0.006	Normal weight	1	
Overweight		79 (37.8)		113 (36.2)				1.64	1.04–2.59
Obesity		86 (41.1)		96 (30.8)				2.1	1.33–3.33
Allergy (pollen or medications)	221								
Yes		97 (43.9)							
No		124 (56.1)							
ICS (mg/day)	221								
		320 (160, 640)							
OCS (dose/year)	221								
Yes		139 (62.9)							
No		82 (37.1)							
%FEV1	191								
		74.8 ± 23.5							
Exacerbations/year	218								
Yes		78 (35.8)							
No		140 (64.2)							
Eosinophils (cells/μL)	213								
		280 (120, 560)							

Quantitative variables: Normal distribution: mean ± standard deviation. Non-normal distribution: P_50_ [P_25_, P_75_]; Qualitative variables: number (percentage); * *p* value for *t* test; N means the whole number of patients considered; n means the number of patients in subgroups; Shade indicates that the *t*-test value is significant (*p* < 0.05); ICS: inhaled corticosteroids; BMI: body mass index; OCS: oral corticosteroids; %FEV1: maximum percentage expiratory volume in the first second of forced expiration.

**Table 3:** Effect of the Cdx2 (rs11568820) polymorphism on susceptibility to asthma.

Models	Genotype	Cases [n (%)]	Controls [n (%)]	*p*-Value ^a^	Adjusted *p*-Value ^b^	OR ^c^	CI 95%
Genotypic	GG	113 (51.4)	232 (54.7)	0.003	0.039	1	
	AG	81 (36.8)	172 (40.6)			2.76	1.46–5.29
	AA	26 (11.8)	20 (4.7)			2.67	1.43–5.04
Dominant	A	107 (48.6)	192 (45.3)	0.418	1		
	GG	113 (51.4)	232 (54.7)				
Recessive	AA	26 (11.8)	20 (4.7)	<0.001	0.011	2.71	1.48–5.02
	G	194 (88.2)	404 (95.3)				
Allelic	A	133 (30.2)	212 (25)	0.045	0.579		
	G	307 (69.8)	636 (75)				
Additive	-	-	-	0.044	0.577		

Shade means the value is significant; OR: odds ratio; CI: confidence interval; ^a^ *p*-value for χ^2^-test; ^b^ *p*-value for Bonferroni correction; ^c^ Unadjusted or crude ORs.

**Table 4:** Impact of clinical characteristics and Cdx2 (rs11568820) polymorphism on susceptibility to asthma.

	Genotypic						Dominant			Recessive			Additive		
	AA vs. GG			AG vs. GG			A vs. GG			AA vs. G			A vs. G		
	*p*-Value	OR	95% CI	*p*-Value	OR	95% CI	*P*-Value	OR	95% CI	*p*-Value	OR	95% CI	*p*-Value	OR	95% CI
BMI															
Overweight	0.017	1.76	1.11–2.80	0.017	1.76	1.11–2.80	0.019	1.73	1.10–2.76	0.017	1.76	1.11–2.80	0.001	1.73	1.10–2.75
Obese	0.001	2.17	1.37–3.48	0.001	2.17	1.37–3.48	0.001	2.17	1.37–3.45	0.001	2.17	1.37–3.48	0.020	2.14	1.36–3.42
rs11568820	0.005	2.73	1.36–5.67	0.994	1.00	0.68–1.46	0.367	1.18	0.82–1.69	0.004	2.73	1.39–5.57	0.051	1.32	1.00–1.75

Shade indicates that the *t*-test value is significant (*p* < 0.05).

**Table 5:** Haplotype association with susceptibility to asthma.

	rs1544410	rs7975232	rs731236	rs4646536	rs703842	rs3782130	rs10877012	Freq.	OR (95% CI)	*p* Value
1	G	C	T	A	T	G	G	0.3329	1.00	---
2	A	A	C	A	T	G	G	0.2382	1.02 (0.69–1.50)	0.93
3	A	A	C	G	C	C	T	0.108	1.30 (0.79–2.15)	0.3
4	G	A	T	A	T	G	G	0.0629	1.79 (0.27–1.19)	0.14
5	G	C	T	G	C	C	T	0.0602	0.63 (0.29–1.35)	0.24
6	A	C	T	A	T	G	G	0.032	2.84 (1.06–7.64)	0.039
7	G	A	T	G	C	C	T	0.0279	0.77 (0.32–1.84)	0.56
8	A	A	T	A	T	G	G	0.0139	1.47 (0.40–5.41)	0.56
9	G	A	C	A	T	G	G	0.0102	2.76 (0.45–17.14)	0.28
rare	*	*	*	*	*	*	*	0.1138	1.40 (0.89–2.22)	0.15

Overall haplotype association p value: 0.031; Freq: haplotype frequency; Shading indicates that the value is significant (*p* < 0.05); * Reference to rare haplotypes as there is no symbol to identify the group.

## Data Availability

Not applicable.

## Supplementary Materials

The following supporting information can be downloaded at: https://www.mdpi.com/article/10.3390/nu15040823/s1, Table S1: Hardy-Weinberg equilibrium; Table S2: Linkage disequilibrium; Table S3: Minor allele frequencies of SNPs; Table S4: Estimation of haplotype frequencies; Table S5: Polymorphisms and association with risk of asthma.
