# Supplementary material for: Association between Single Nucleotide Polymorphisms Related to Vitamin D Metabolism and the Risk of Developing Asthma
Source: Nutrients. 2023 Feb 5;15(4):823. doi: 10.3390/nu15040823 (PMC9964183; doi:10.3390/nu15040823)
Supplement: Supplementary file 1 [file nutrients-15-00823-s001.zip › nutrients-2170231-supplementary.pdf]

Table S1. Hardy-Weinberg Equilibrium.

Chr	SNP	Sample	Minor Allele	Major Allele	Genotype counts	Observed heterozygosity	Expected heterozygosity	p-value
4	rs7041	ALL	T	G	140/335/178	0.513	0.4983	0.4801
4	rs7041	AFF	T	G	43/109/67	0.4977	0.494	1.00
4	rs7041	UNAFF	T	G	97/226/111	0.5207	0.4995	0.4418
11	rs10741657	ALL	A	G	96/284/271	0.4363	0.4639	0.1288
11	rs10741657	AFF	A	G	36/91/93	0.4136	0.4664	0.111
11	rs10741657	UNAFF	A	G	60/193/178	0.4478	0.4625	0.5321
12	rs731236	ALL	C	T	108/311/233	0.477	0.4816	0.8075
12	rs731236	AFF	C	T	41/100/79	0.4545	0.4851	0.4038
12	rs731236	UNAFF	C	T	67/211/154	0.4884	0.4797	0.7637
12	rs7975232	ALL	C	A	145/341/169	0.5206	0.4993	0.309
12	rs7975232	AFF	C	A	55/108/58	0.4887	0.4999	0.7878
12	rs7975232	UNAFF	C	A	90/233/111	0.5369	0.4988	0.1243
12	rs1544410	ALL	A	G	132/311/211	0.4755	0.4927	0.3829
12	rs1544410	AFF	A	G	43/101/77	0.457	0.4882	0.3375
12	rs1544410	UNAFF	A	G	89/210/134	0.485	0.4946	0.6979
12	rs2228570	ALL	T	C	84/301/269	0.4602	0.46	1.00
12	rs2228570	AFF	T	C	30/91/100	0.4118	0.4498	0.2311
12	rs2228570	UNAFF	T	C	54/210/169	0.485	0.4647	0.4084
12	rs11568820	ALL	A	G	46/253/345	0.3929	0.3922	1.00
12	rs11568820	AFF	A	G	26/81/113	0.3682	0.4218	0.07743
12	rs11568820	UNAFF	A	G	20/172/232	0.4057	0.375	0.1193
12	rs4646536	ALL	G	A	36/264/352	0.4049	0.3826	0.1522
12	rs4646536	AFF	G	A	12/92/117	0.4163	0.3871	0.3015
12	rs4646536	UNAFF	G	A	24/172/235	0.3991	0.3802	0.3746
12	rs3782130	ALL	C	G	34/264/354	0.4049	0.3796	0.09895
12	rs3782130	AFF	C	G	12/92/115	0.4201	0.3894	0.2991
12	rs3782130	UNAFF	C	G	22/172/239	0.3972	0.3744	0.2479
12	rs10877012	ALL	T	G	36/259/358	0.3966	0.3784	0.2552
12	rs10877012	AFF	T	G	14/93/114	0.4208	0.3976	0.4981
12	rs10877012	UNAFF	T	G	22/166/244	0.3843	0.368	0.4325
12	rs703842	ALL	C	T	35/264/350	0.4068	0.3822	0.123
12	rs703842	AFF	C	T	13/95/112	0.4318	0.3988	0.309
12	rs703842	UNAFF	C	T	22/169/238	0.3939	0.3732	0.3008
20	rs4809957	ALL	G	A	21/246/375	0.3832	0.348	0.01218
20	rs4809957	AFF	G	A	8/84/127	0.3836	0.3524	0.2499
20	rs4809957	UNAFF	G	A	13/162/248	0.383	0.3457	0.0339
20	rs6068816	ALL	T	C	11/135/506	0.2071	0.2118	0.5775
20	rs6068816	AFF	T	C	4/43/174	0.1946	0.2041	0.5035
20	rs6068816	UNAFF	T	C	7/92/332	0.2135	0.2157	0.8226

Chr: Chromosome; ALL: All population; AFF: Case group; UNAFF: Control group
Shade means the value is significant for t test (p<0.05).

Table S2. Linkage disequilibrium

Chr	BP	SNP	Chr	BP	SNP	R2	D’

12	47844974	rs731236	12	47845054	rs7975232	0.578315	-
12	47844974	rs731236	12	47846052	rs1544410	0.562537	-
12	47845054	rs7975232	12	47846052	rs1544410	0.402096	0.792282
12	57764205	rs4646536	12	57768115	rs3782130	0.810826	0.924972
12	57764205	rs4646536	12	57768302	rs10877012	0.673952	0.868872
12	57764205	rs4646536	12	57768956	rs703842	0.754507	0.899834
12	57768115	rs3782130	12	57768302	rs10877012	0.706778	0.874134
12	57768115	rs3782130	12	57768956	rs703842	0.798471	0.908186
12	57768302	rs10877012	12	57768956	rs703842	0.64727	0.851107
Chr: Chromosome; BP: Physical position (base-pair)							

Table S3. Minor allele frequencies of SNPs.

Chr	SNP	Minor Allele	Major Alelle	MAF
4	rs7041	T	G	0.4709
11	rs10741657	A	G	0.3656
12	rs731236	C	T	0.4041
12	rs7975232	C	A	0.4817
12	rs1544410	A	G	0.4396
12	rs2228570	T	C	0.3586
12	rs11568820	A	G	0.2679
12	rs4646536	G	A	0.2577
12	rs3782130	C	G	0.2546
12	rs10877012	T	G	0.2534
12	rs703842	C	T	0.2573
20	rs4809957	G	A	0.2243
20	rs6068816	T	C	0.1204
Chr: Chromosome; MAF: Minor allele frequency				

Table S4. Haplotype frequencies estimation.

	rs1544410	rs7975232	rs731236	rs4646536	rs703842	rs3782130	rs10877012	Total	Case group	Control group	Cumulative frequency
1	G	C	T	A	T	G	G	0.3338	0.3606	0.3197	0.3338
2	A	A	C	A	T	G	G	0.2393	0.2535	0.2323	0.5731
3	A	A	C	G	C	C	T	0.1055	0.0997	0.1086	0.6786
4	G	C	T	G	C	C	T	0.0636	0.0834	0.0534	0.7422
5	G	A	T	A	T	G	G	0.0626	0.0508	0.0684	0.8049
6	A	C	T	A	T	G	G	0.031	0.0123	0.0416	0.8359
7	G	A	T	G	C	C	T	0.0254	0.0343	0.02	0.8613
8	A	A	T	A	T	G	G	0.0138	0.0095	0.016	0.8751
9	G	A	C	A	T	G	G	0.013	0.0061	0.0164	0.888
10	G	A	C	G	C	C	T	0.0076	0.0125	0.0052	0.8956
11	A	C	T	G	C	C	T	0.0072	0.0036	0.0086	0.9028
12	A	A	C	G	C	C	G	0.007	0.0022	0.0093	0.9098
13	G	C	T	A	T	G	T	0.0062	0.0029	0.0086	0.9161
14	G	C	T	G	C	C	G	0.0061	NA	0.0098	0.9222
15	G	C	T	A	C	G	G	0.0057	0.0069	0.0033	0.9279
16	G	A	T	A	T	G	T	0.0057	NA	0.01	0.9336
17	G	C	T	A	C	C	T	0.0054	0.0114	0.0023	0.939
18	G	C	T	G	T	G	G	0.0052	0.0046	0.0056	0.9442
19	A	A	C	G	T	C	T	0.0049	0.0092	0.0033	0.949
20	A	A	C	A	C	G	G	0.0048	0.0033	0.0064	0.9538
21	A	A	T	G	C	C	T	0.0047	NA	0.0076	0.9586
22	G	C	T	A	T	C	G	0.0044	0	0.0058	0.9629
23	A	A	C	A	T	G	T	0.004	0.0054	0.0019	0.9669
24	A	A	C	G	C	G	G	0.0029	0.0046	0.0017	0.9699
25	G	C	T	G	T	C	T	0.0025	NA	0.003	0.9724
26	A	C	C	A	T	G	G	0.0025	0.0035	0.001	0.9749
27	G	C	T	G	C	G	T	0.002	NA	0.0036	0.9769
28	A	A	C	G	T	C	G	0.0019	0.0023	0.0023	0.9788
29	G	A	T	G	T	C	T	0.0019	NA	0.0031	0.9807
30	A	A	C	G	T	G	G	0.0019	NA	0.0023	0.9825
31	G	A	T	G	C	C	G	0.0017	NA	0.0024	0.9842
32	G	C	C	A	T	G	G	0.0017	NA	0.0029	0.9859
33	A	A	T	A	T	G	T	0.0014	0.0023	NA	0.9873
34	A	A	C	A	T	C	G	0.0012	0.0023	0.0015	0.9885
35	G	C	T	G	C	G	G	0.001	NA	0.0027	0.9895
36	A	C	C	G	C	C	T	0.001	0.004	NA	0.9904
37	A	A	C	A	C	G	T	9e-04	0.003	NA	0.9913
38	A	A	C	A	C	C	G	8e-04	0.0023	NA	0.9921
39	A	C	C	A	T	C	G	8e-04	NA	0.0012	0.9929
40	G	C	C	G	T	G	G	8e-04	NA	0.0012	0.9937
41	G	A	T	G	T	G	T	8e-04	NA	0.0012	0.9945
42	A	A	T	A	T	C	G	8e-04	NA	0.0012	0.9952
43	G	C	C	G	T	G	T	8e-04	NA	0.0012	0.996
44	G	A	T	A	C	G	T	7e-04	NA	0.0011	0.9967
45	G	A	T	G	C	G	T	6e-04	0.0023	NA	0.9973
46	G	A	C	A	T	C	G	6e-04	NA	9e-04	0.9979
47	G	A	T	G	C	G	G	6e-04	NA	NA	0.9985
48	A	A	T	A	C	G	G	5e-04	NA	0.001	0.9991
49	A	A	C	G	C	G	T	5e-04	NA	0	0.9996
50	A	A	T	G	T	G	G	4e-04	NA	4e-04	1
51	G	A	T	A	T	C	G	0	NA	NA	1
52	G	A	T	A	C	G	G	0	0.0013	NA	1

* Rare haplotypes; NA: not available

Table S5. Polymorphisms and association with risk of asthma.

SNP	Minor Allele	Mayor Allele	Model	Cases	Controls	χ²	p-value χ²	p-value Fisher	Adjusted p-value*
rs7041	T	G	Genotypic	43/109/67	97/226/111	1.995	0.3687	0.3793	1
			Additive	195/243	420/448	1.8	0.1797	0.1797	1
			Allelic	195/243	420/448	1.747	0.1863	0.1967	1
			Dominant	152/67	323/111	1.848	0.174	0.1926	1
			Recessive	43/176	97/337	0.6373	0.4247	0.4798	1
rs10741657	A	G	Genotypic	36/91/93	60/193/178	1.012	0.6029	0.6032	1
			Additive	163/277	313/549	0.06396	0.8003	0.8003	1
			Allelic	163/277	313/549	0.06777	0.7946	0.8079	1
			Dominant	127/93	253/178	0.0568	0.8116	0.8665	1
			Recessive	36/184	60/371	0.6912	0.4058	0.415	1
rs731236	C	T	Genotypic	41/100/79	67/211/154	1.214	0.545	0.5368	1
			Additive	182/258	345/519	0.2463	0.6197	0.6197	1
			Allelic	182/258	345/519	0.2486	0.618	0.6333	1
			Dominant	141/79	278/154	0.004322	0.9476	1	1
			Recessive	41/179	67/365	1.031	0.3098	0.3176	1
rs7975232	C	A	Genotypic	55/108/58	90/233/111	1.817	0.4031	0.3971	1
			Additive	218/224	413/455	0.3712	0.5423	0.5423	1
			Allelic	218/224	413/455	0.3554	0.5511	0.5592	1
			Dominant	163/58	323/111	0.03416	0.8534	0.8508	1
			Recessive	55/166	90/344	1.463	0.2265	0.2335	1
rs1544410	A	G	Genotypic	43/101/77	89/210/134	1.016	0.6017	0.6072	1
			Additive	187/255	388/478	0.7151	0.3977	0.3977	1
			Allelic	187/255	388/478	0.7401	0.3896	0.4099	1
			Dominant	144/77	299/134	1.016	0.3136	0.3312	1
			Recessive	43/178	89/344	0.1094	0.7409	0.7586	1
rs2228570		G	Genotypic	30/91/100	54/210/169	3.219	0.2	0.1987	1
			Additive	151/291	318/548	0.8328	0.3615	0.3615	1
			Allelic	151/291	318/548	0.8323	0.3616	0.3935	1
			Dominant	121/100	264/169	2.337	0.1263	0.1313	1
			Recessive	30/191	54/379	0.1592	0.6899	0.7116	1
rs11568820	A	G	Genotypic	26/81/113	20/172/232	11.05	0.003991	0.00527	0.039
			Additive	133/307	212/636	4.043	0.04436	0.04436	0.5767
			Allelic	133/307	212/636	4.036	0.04453	0.04674	0.5789
			Dominant	107/113	192/232	0.6548	0.4184	0.4536	1
			Recessive	26/194	20/404	11.01	0.0009051	0.00183	0.01086
rs4646536	G	A	Genotypic	12/92/117	24/172/235	0.1799	0.914	0.9156	1
			Additive	116/326	220/642	0.08464	0.7711	0.7711	1
			Allelic	116/326	220/642	0.07969	0.7777	0.7893	1
			Dominant	104/117	196/235	0.1474	0.701	0.74	1
			Recessive	12/209	24/407	0.005378	0.9415	1	1
rs3782130	C	G	Genotypic	12/92/115	22/172/239	0.4252	0.8085	0.784	1
			Additive	116/322	216/650	0.3904	0.5321	0.5321	1
			Allelic	116/322	216/650	0.3643	0.5461	0.5455	1
			Dominant	104/115	194/239	0.4225	0.5157	0.5603	1
			Recessive	12/207	22/411	0.04676	0.8288	0.853	1
rs10877012	T	G	Genotypic	14/93/114	22/166/244	1.542	0.4627	0.4439	1
			Additive	121/321	210/654	1.53	0.2161	0.2161	1
			Allelic	121/321	210/654	1.457	0.2275	0.2272	1
			Dominant	107/114	188/244	1.416	0.2341	0.2454	1
			Recessive	14/207	22/410	0.4331	0.5105	0.5871	1
rs703842	C	T	Genotypic	13/95/112	22/169/238	1.24	0.5379	0.5223	1
			Additive	121/319	213/645	1.164	0.2807	0.2807	1
			Allelic	121/319	213/645	1.089	0.2967	0.3145	1
			Dominant	108/112	191/238	1.222	0.269	0.2803	1
			Recessive	13/207	22/407	0.1738	0.6768	0.7147	1
rs4809957	G	A	Genotypic	8/84/127	13/162/248	0.1584	0.9238	0.9382	1

			Additive	100/338	188/658	0.0684	0.7937	0.7937	1
			Allelic	100/338	188/658	0.06148	0.8042	0.8324	1
			Dominant	92/127	175/248	0.02418	0.8764	0.9327	1
			Recessive	8/211	13/410	0.1532	0.6955	0.8154	1
rs6068816	T	C	Genotypic	4/43/174	7/92/332	NA	NA	0.8135	NA
			Additive	51/391	106/756	0.1553	0.6936	0.6936	0.4109
			Allelic	51/391	106/756	0.1587	0.6903	0.7199	0.3761
			Dominant	47/174	99/332	NA	NA	0.6915	NA
			Recessive	4/217	7/424	NA	NA	1	NA
Chr: chromosome; NA: not applicable; * p-value for Bonferroni correction<br>Shade means the value significant for t test (p<0.05)									
